# The complex interplay between perception, cognition, and action: a commentary on Bach et al. 2022

**DOI:** 10.1007/s00426-023-01921-w

**Published:** 2024-01-31

**Authors:** Helen O’Shea, Judith Bek

**Affiliations:** 1 School of Psychology, University College Dublin, Belfield, Dublin 4 Ireland; 2 Faculty of Kinesiology and Physical Education, University of Toronto, Toronto, Canada

## Abstract

Bach (Psychological Research 2022, https://doi.org/10.1007/s00426-022-01773-w) offer a re-conceptualisation of motor imagery, influenced by older ideas of ideomotor action and formulated in terms of action effects rather than motor output. We share the view of an essential role of action effect in action planning and motor imagery processes, but we challenge the claim that motor imagery is non-motoric in nature. In the present article, we critically review some of Bach et al.’s proposed ideas and pose questions of whether effect and motor processes are functionally separable, and if not, what mechanisms underlie motor imagery and what terminology best captures its function.

In their article, Bach et al. (2022) adopt a novel position in relation to the fundamental mechanisms underlying motor imagery (MI). They suggest that the process of imagining actions (i.e., MI) is formulated in terms of action effects rather than motor output, a proposal based on the traditional ideomotor theory (i.e., where actions are internally represented via the desired action effects, e.g., Carpenter, 1852; Greenwald, 1970). Bach et al. propose that effect-based action planning and control as it purportedly ensues during action execution, is a central process of MI, with little if any need for motoric processes. Given that no single comprehensive theory of motor cognition exists, this paper is a welcome addition to the ongoing discussion about whether certain action-related functions are the best conceptualised as motoric or non-motoric. Indeed, a strength of the paper is that it highlights the persistent problem of an underspecified theoretical basis of MI. Currently, different theories of action-related processes (including MI) exist, but while an overarching theme of these is the interconnection between perceptual-motor-cognitive systems, there is less consensus around the fundamental mechanisms driving MI, action planning, and action control (for review, see Hurst & Boe, 2022; Jeannerod, 2001, 2006; Grush, 2004; Shin et al., 2023; Hommel, 2019; Glover & Baran, 2017; Wolpert & Ghahramani, 2000; Hesslow, 2012). We share the view expressed by Bach et al. of an essential role of action effects in MI processes, but also consider MI to be fundamentally motor-cognitive rather than perceptual, for which we set out our reasoning below.

A key question arising from Bach et al.’s account of MI is whether an effect-based conceptualisation offers anything radically different from existing representational explanations of MI-related action processes. For instance, motor simulation theory (MST; Jeannerod, 2001, 2006) and emulation theory (Grush, 2004) highlight the key role of anticipating sensory consequences (Jeannerod, 2006) and sensory feedback (Grush, 2004) in the generation and adaptation of accurate motor signals during MI (and during motor execution). According to these motor-based theories of MI, MI relies in part on predicted effects for action representation and simulation, and so these effects are integral to the formation of action representations.

In this regard, instances (mentioned by Bach et al.) where sensory effects prime motor performance (e.g., Land, 2018) do not especially differentiate effect-based control from simulation-based or forward-modeling accounts, because priming may arise from action−effect association or from fast and automatic simulation and anticipation of sensory effects via efference copy routes, respectively (Hommel, 2019; Jeannerod, 2006; Wolpert & Ghahramani, 2000). It is also worth noting that evidence indicates that behavioural or neural priming of motor systems can occur with mere abstract amodal ideas (e.g., categories “long” vs “short” can prime long vs short movement; Shin et al., 2023) and with abstract language (Sakreida et al., 2013), respectively. Given this priming influence of abstract information on activity in neural motor systems, it is evident that neural motor activation does not merely involve implicit effect-based association (Harpaintner et al., 2020), but that additional mediating processes can occur, and these can have some level of conceptual processing and some level of pragmatic motor processing (Shin et al., 2023).

In relation to the aforementioned efference copy routes, an important point raised by the authors in criticising “standard accounts” of MI, is that “imagery emerges from motor commands being fed to a forward model-like mechanism …[but] where [do] these motor commands come from in the first place” and “why is it necessary to identify the precise motor command that brings about this imagination” and “…fool [efference copy] mechanisms into completing the same job for actions that one does decidedly not want to execute” (Bach et al., 2022, p.5). To unpack this issue, it is important to note that according to computational motor control theory and MST, action representations exploit efference copy routes because they offer detached (more cognitive) motor pathways to rehearse or simulate the represented action (Jeannerod, 2006; Wolpert & Ghahramani, 2000). In this regard, neurophysiological evidence shows that during delayed reaching, preparatory and generative neurocognitive aspects of movement are orthogonal in nature (i.e., poorly correlated, although not fully uncorrelated) thereby positioning action-related processes across different dimensions that likely facilitate different computations—whether motor output or (more cognitive) efferent copy/simulation as might occur during MI (Ames et al., 2019; Kaufman et al., 2014). Indeed, if higher-level action-planning representation comprises abstract perceptual and motor codes, each weighted according to the current context and goal (as theory of event coding claims; Hommel, 2019), this more abstract action representation may then give way to more specific processes relating to movement production (Churchland et al., 2010; Schween et al., 2019), whether via efferent output (execution) or efference copy (MI) processes.

Efference copy or simulation is purportedly the very mechanism that allows effects and actions to be linked, activating both perceptual and motor pathways during the representational process, and facilitating anticipation of action consequences (Wolpert & Ghahramani, 2000). Accordingly, this alternative motor (efference copy) route has the capacity to alter action representations and offers a mechanism for adaptation or improvement in execution and for performance of MI. For example, the research shows that individuals who physically execute the same initial reaching movement in a movement sequence, but use MI to follow-through with two different subsequent movements, can learn two different movements and form the associated two new motor memories (Sheahan et al., 2018). Here, the same initial physical limb state provides experience of relatively stable sensory effects and only MI is used to learn the second stage of each movement. Accordingly, although Bach et al. propose that MI relates to effect-related processes, the new movement sequences learned in this instance have not previously been executed, and so, the participants have not had the opportunity to “have done them over and over again …and internalise how they look …feel” to “simply recall [the] perceptual knowledge to produce a vivid experience of the actions they want to imagine” (p.4). Thus, it seems that MI exploits at least some motoric processes in acquiring the new motor skill.

In conclusion, Bach et al.’s article offers a refreshing perspective on MI and the complex interplay between perception, cognition, and motor functions. However, given the overlap between this effect-based account and existing conceptualisations of MI, in terms of a fundamental involvement of effect information in informing action processes, we suggest that comprehensive theorising about action-related processes should delineate both the content and the functional mechanisms of action representations across tasks and contexts. In this regard, a fundamental consideration is not only the integration of action and effect codes in the action plan but also the hierarchical or dimensional nature of processing action information across different action processes and types. Processing of action unfolds over time and so it seems important to uncover the relative contribution of perceptual−motor−cognitive systems along this continuum rather than seeking categorical distinctions (action planning versus action execution; MI versus execution) that are functionally separable. Such categorical demarcations may restrict progress in understanding fundamental links between perceptual, motor, and cognitive functions. Overall, while we consider perceptual effects an important and even essential component of MI, we also consider them only part of the action representation and simulation process and refute the notion that MI comprises no motoric element.

## Data Availability

Not applicable.
